# Modelling Terahertz Wave Generation in InAs Quantum-Dot Containing GaAs-Based Photoconductive Antenna

**DOI:** 10.3390/nano16201291

**Published:** 2026-10-12

**Authors:** Osamu Wada, Yasuo Minami, Takahiro Kitada, Toshiyuki Kaizu, Yukihiro Harada, Takashi Kita

**Affiliations:** 1 Office for Academic and Industrial Innovations (Oacis), Kobe University, 1-1 Rokkodai, Nada, Kobe 657-8501, Hyogo, Japan; 2 College of Industrial Technology, Nihon University, 1-2-1 Izumi-cho, Narashino 275-8575, Chiba, Japan; 3 Department of Electrical Engineering and Computer Science, National Institute of Technology, Matsue College, 14-4 Nishi-Ikumacho, Matsue 690-8518, Shimane, Japan; 4 Quantum Future Creative Device Development Center, The University of Electro-Communications, 1-5-1 Chofugaoka, Chofu 181-8585, Tokyo, Japan; 5 Graduate School of Engineering, Kobe University, 1-1 Rokkodai, Nada, Kobe 657-8501, Hyogo, Japan

**Keywords:** THz wave generation, GaAs-based photoconductive antenna (PCA), InAs/GaAs quantum dots, midgap states, rate equations, space charge Coulomb screening, THz field screening, quantum dot capture time for electrons

## Abstract

InAs quantum-dot-containing GaAs-based photoconductive antenna (PCA) devices are promising for terahertz (THz) generation under below-bandgap excitation by ultrafast laser pulses. This paper describes the results of rate equation analyses of these devices by assuming the two-step photocarrier generation via midgap states in GaAs and carrier capture into the quantum dots. In the present simulation model, the Coulomb space charge screening effect and the radiated THz field screening effect are taken into consideration. The calculations indicate that, at low excitation powers, the radiated THz field is primarily governed by the fast photocurrent transient arising from rapid electron capture into the quantum dots. At higher photoexcitation power, the THz radiation is controlled predominantly by the carrier velocity reduction immediately after the excitation pulse due to the Coulomb space charge screening effect. As the photoexcitation power increases, the Coulomb screening effect causes saturation of the THz field amplitude, whereas the radiated THz field screening effect contributes little to the saturation of the THz field amplitude. In this study, the influence of key parameters, such as the density and electron capture time of the midgap states and quantum dots, are analyzed. Existing experimental data on THz emission characteristics are discussed on the basis of the present analysis. The observed features provide comprehensive understanding on the THz generation process involving many physical parameters in PCAs and can be applied to optimizing design for high-performance devices.

## 1. Introduction

Terahertz (THz) radiation in the 0.1–10 THz range is used in a wide variety of fields, including security screening, environmental sensing, imaging [1,2], telecommunications [3,4], and spectroscopic measurement systems [5,6,7]. A variety of THz wave generation and detection devices have been developed by using compound semiconductor materials, including photoconductive antennas (PCAs) [8,9,10,11], quantum cascade lasers (QCLs) [12,13], resonant tunneling diodes [14,15], and photodiode-based photomixers [16,17]. Among those devices, PCAs are particularly useful since both the generation and detection of THz wave in a broad spectral range are possible by the simple combination with the conventional ultrafast pulse laser, and they have been widely used in practical THz time-domain spectroscopy (TDS) systems. Their advantages of high mobility and short lifetime of the photocarriers in semiconductors merit high performance in THz emission and detection. The most popular PCAs consist of lateral photoconductors using low-temperature grown GaAs (LT-GaAs) [8,9] which are photoexcited at the wavelength above the GaAs bandgap wavelength (870 nm). However, PCAs operating at the telecom wavelength (particularly at 1550 nm) are required for wider applications of THz systems with high stability, compactness, and low cost. Numbers of studies have been conducted on PCAs with low bandgap materials [18] such as InGaAs [19] and InGaAsBi [20]. Insufficient dark resistivity anticipated for low bandgap materials is challenged by various methods such as the dopant [21] and quantum dot (QD) [22] inclusion and band engineering of the compositional structures [23,24,25,26]. As another approach for the telecom wavelength operation of PCAs, the two-step excitation via midgap states (e.g., EL2-like states) in GaAs has been investigated by several research groups [27,28,29,30]. Such GaAs-based structures are advantageous to avoiding the dark current problem, owing to the large bandgap energy of GaAs.

The incorporation of QDs in GaAs-based PCAs was first demonstrated at the excitation in 800 nm band [31,32], and its most significant advantage is in the shortening of photocarrier lifetime [33]. The lifetime shortening effects have been reported to contribute to the THz generation in InAs/GaAs QD PCA under 800 nm photoexcitation [34] and more recently in Er-InAs QD containing InGaAs PCA under 1500 nm photoexcitation [35]. On a basis of these results, we have recently proposed and demonstrated the THz generation from InAs QD-containing GaAs PCAs under photoexcitation at 1550 nm [36]. This structure combines the advantages of 1550 nm excitation, low dark current inherent to GaAs, and lifetime shortening introduced by QDs. Basic photoconductivity characteristics of this material were studied in our recent work [37]. However, the detailed understanding of the THz generation process in such materials has not yet been achieved.

To fully understand the physical processes in the THz generation and to improve the structural optimization of PCA devices, it is most important to develop a comprehensive model of THz generation in QD-containing GaAs-based PCAs. There have been a number of theoretical and experimental studies published on LT-GaAs- and GaAs-based PCAs, which include discussions on the below-bandgap photoexcitation through two-step excitation [32,33,34,35], carrier capture by trap states and their effects on the THz spectra [38] and various photocarrier-induced screening effects and their influence on the THz radiation amplitude [39,40,41]. However, modelling of the THz generation in QD-containing GaAs PCAs with below-GaAs bandgap excitation has not been investigated so far. Therefore, in this paper we present the development of a simplified model of THz generation in InAs QD-containing GaAs-based PCAs under below-bandgap photoexcitation. On the basis of this model, the major physical processes including ultrafast photocarrier dynamics, space charge- and radiated field-screening effects, and the role of QDs in these processes are discussed. In the following, we first describe briefly the PCA material and structure which we have used in our prior work and then explain theoretical formulae used for our model. The results of simulation and characterization of THz generation properties are presented and discussed.

## 2. Material and Device Structure

In this study, we assume the QD-containing material and PCA device structure are the same as those used in our earlier works [36,37], and their basic features are summarized in this section. The material consists of a serial layer grown on a semi-insulating (SI) GaAs (001) substrate by using molecular beam epitaxy (MBE) [42]. The layer sequence includes a 150-nm-thick undoped GaAs buffer layer, a set of stacked 20 periods of QD/spacer pair which consists of self-assembled InAs QDs with a nominal thickness of 2.0 monolayers (ML) and a 50-nm-thick undoped GaAs spacer layer, and a 30-nm-thick undoped LT-GaAs layer as the capping layer. The high resistivity of LT-GaAs layer is ensured through the post growth rapid thermal annealing. The total thickness of the GaAs layers (excluding the buffer layer), which form the carrier transport layer, is 1180 nm, and high-quality transport properties are maintained in the whole GaAs transport layer. The QD density is estimated to be 1.7 × 10^15^ cm^−3^ as determined from the atomic force microscope evaluation of surface density of QDs (1 × 10^10^ cm^−2^) [42]. The QD sub-band wavelength is ~1200 nm. No wavefunction couplings in either vertical or horizontal directions are anticipated due to large interdot spacings.

We assume a PCA device structure having an active region of 6 μm length and 10 μm width, which is fabricated by mesa etching (to minimize the dark current) followed by Ti/Au contact deposition [42]. THz wave generation properties are characterized using standard optics including the photoexcitation by a femtosecond laser pulse at the wavelength of 1550 nm. The THz wave is extracted from the rear surface of the PCA through a Si lens and detected by another LT-GaAs-based commercial PCA excited by the idler beam (~800 nm) of the laser source [36].

## 3. Simulation Model

The approach we adopted for modeling the photocurrent dynamics in the QD-containing GaAs PCA device is based on the rate equation analysis using the Drude–Lorentz model [43,44,45,46,47]. The photocarrier generation is performed through the two-step photoexcitation process via midgap states included in the GaAs transport layer under the below-bandgap photoexcitation at 1550 nm [22,36]. Given that the electron mobility (6000–8000 cm^2^/(Vs)) in undoped GaAs is more than an order of magnitude higher than the hole mobility (300–400 cm^2^/(Vs)) [48], the hole contribution to carrier transport is neglected in the present analysis. The temporal variation of the electron density in the GaAs transport layer can be expressed by the following rate equations:

(1) $$\frac{dn}{dt}=U\left(t\right)-\frac{n}{\begin{array}{c} \tau_{cT} \end{array}}\left(\begin{array}{c} 1-\frac{n_{T}}{\begin{array}{c} N_{T} \end{array}} \end{array}\right)-\frac{n}{\begin{array}{c} \tau_{cQ} \end{array}}\left(1-\frac{\begin{array}{c} n_{Q} \end{array}}{\begin{array}{c} N_{Q} \end{array}}\right),$$

(2) $$\frac{dn_{T}}{dt}=-U\left(t\right)+\frac{n}{\tau_{cT}}\left(1-\frac{n_{T}}{N_{T}}\right),$$

(3) $$\frac{dn_{Q}}{dt}=\frac{n}{\tau_{cQ}}\left(1-\frac{n_{Q}}{N_{Q}}\right),$$

where *n* is the density of electrons in the conduction band of the transport layer, which partially includes the wetting layers and LT-GaAs capping layer; *U*(*t*) is the generation rate of photoelectrons; *n*_T_ and *n*_Q_ are the electron densities occupying the midgap states and the QDs; *N*_T_ and *N*_Q_ are the total electron densities of states for the midgap states and the QDs; and *τ*_cT_ and *τ*_cQ_ are the capturing times for electrons from the conduction band into the midgap states and into the QDs. Since we are primarily interested in the 1 ps time scale, the electron–hole recombination process in the GaAs transport layer is neglected in the present analysis. The recombination lifetime in GaAs under strong ultrafast laser excitation is estimated to be in the range from 10 ps to 100 ps [49] or from 90 ps to 300 ps [50], which is sufficiently longer than the time scale considered here. The thermal excitation of electrons from the midgap states and QDs is also neglected. The electron escape time from the ground states of InAs QDs, which are shallower than the midgap states, to the GaAs conduction band has been reported to range from 50 to 200 ps [51]. In Equations (2) and (3), we consider the case *n*_T_ ≤ *N*_T_ and *n*_Q_ ≤ *N*_Q_, where the midgap states or the QDs are not fully occupied with the electrons. Assuming a Gaussian profile of the excitation laser pulse, the generation rate of electrons *U*(*t*) is determined by

(4) $$U\left(t\right)=\frac{{I_{0}\sigma }_{TB}}{hc/\lambda }n_{T0}\exp\left(-\frac{t^{2}}{w^{2}}\right)\left(1-R\right),\;$$

where *I*_0_ is the peak power density of the excitation laser pulse on the PCA surface, *σ*_TB_ is the cross-section for the electron transition from the midgap state to the conduction band, *h* is the Planck constant, *c* is the light velocity, *λ* is the wavelength, *w* is the width of the laser pulse, *R* is the surface reflectivity the PCA device, and *n*_T0_ is the steady state value of the *n*_T_ before the excitation pulse arrival. For a Gaussian pulse, the value of *w* denoted in Equation (5) is correlated with the full-width at half-maximum (FWHM) of the laser pulse by $w=\frac{FWHM}{2{\left(ln2\right)}^{1/2}}=\frac{FWHM}{1.665}$. The value of peak power density of laser pulse *I*_0_ is determined by $I_{0}=\frac{P_{ex}}{wf_{rep}}$, where *P*_ex_ is the averaged laser pulse power density, and *f*_rep_ is the pulse repetition rate [47].

The photogenerated electrons are accelerated by the bias electric field in the transport layer. The temporal change of the velocity of the electrons is expressed in the equation of motion of electrons as [43]

(5) $$\frac{dv}{dt}=-\frac{\begin{array}{c} v \end{array}}{\begin{array}{c} \tau_{s} \end{array}}+\frac{e}{\begin{array}{c} m^{*} \end{array}}E_{in},\;$$

where *v* is the average velocity of electrons, *τ*_s_ is the electron momentum relaxation time, *e* is the unit charge, and m^*^ is the electron effective mass. *E*_in_ is the internal field, which is the electric field at the position of carrier electron, and is evaluated by taking account of the Coulomb screening effect caused by the separating carriers as shown in the following expression:

(6) $$E_{in}=E_{b}-\frac{P}{\eta \varepsilon },\;$$

where *E*_b_ is the externally applied bias field, *P* is the space charge polarization created by the spatial separation of electron and hole, and *ε* is the dielectric constant (12.9) of GaAs transport layer. The factor 1/*η* is known as the depolarization factor [52] and is equal to 1/3 for microscopically isotropic materials, including GaAs. The temporal variation of the space charge polarization is represented by the ordinary Debye type relaxation

(7) $$\frac{dP}{dt}=-\frac{P}{\begin{array}{c} \tau_{r} \end{array}}+J,\;$$

where *τ*_r_ is the polarization relaxation time, and *J* is the current density produced by the photogenerated electrons. The relaxation time *τ*_r_ is represented by the carrier recombination time, whose contribution is assumed to be negligible in this device, consistent with the assumption described above. Equation (7) thus results in a simple form d*P*/d*t* = *J*. The THz radiation is caused by the current transient according to Maxwell’s equations, and the far-field electric field *E*_THz_ is simply expressed as

(8) $$E_{THz}\propto \frac{dJ}{dt}.\;$$

This expression is obtained by ignoring the effects of the radiation pattern, propagation and reflection losses of the generated THz radiation in the PCA device, as well as the effects of the antenna structure, measurement optics, and detector response characteristics. In this calculation model, we use d*J*/d*t* peak height to represent the fundamental characteristics of the radiated THz field intensity.

To evaluate the temporal change of the current density, another field screening mechanism which is caused by the radiated THz field itself has been investigated [53,54]. If the radiated THz field has a near-field component along with the direction of the bias field, it can limit the output of THz radiation. This mechanism operates most efficiently in a planar device where the bias field is parallel to the current flow. By incorporating this radiated THz field screening, the current density *J* is expressed by [55,56,57]

(9) $$J=\frac{evn}{\begin{array}{c} 1+\frac{evndZ_{0}}{\left(1+n_{dc}\right)E_{in}} \end{array}}=\frac{en\mu_{e}E_{in}}{\begin{array}{c} 1+\frac{en\mu_{e}dZ_{0}}{\begin{array}{c} 1+n_{dc} \end{array}} \end{array}},\;$$

where *μ*_e_ is the electron mobility, *d* is the transport layer thickness, *n*_dc_ is the refractive index (=*ε*^1/2^) of the transport layer at the THz frequency, and *Z*_0_ is the free space impedance (377 Ω). The relation of *v* = *μ*_e_*E*_in_ is used in Equation (9). Note that although the same relationship as Equation (9) is expressed by using the surface current density and surface electron density in the original paper [56], it has been converted to the volume current density and volume carrier density here. Also, the bias field applied to the PCA in [56] has been replaced by the internal field *E*_in_, which involves the effect of Coulomb screening so that both the Coulomb screening and radiated THz field screening have been taken account in Equation (9).

To analyze the temporal behavior of various parameters in the transport layer, a set of differential Equations (1)–(3), (5) and (7) is numerically solved by using the fourth order Runge–Kutta method. In the calculation, we use the excitation laser pulse with the wavelength *λ* of 1550 nm, FWHM width of 100 fs, and the repetition rate of 80 MHz. The electron effective mass in GaAs m^*^ is 0.067 *m*_0_, where *m*_0_ is the free electron mass (9.109 × 10^−31^ kg), and the electron momentum relaxation time is assumed to be 200 fs, which leads to the steady-state electron mobility *μ*_e_ = *eτ*_s_/m^*^ = 5200 cm^2^/(Vs). This is in reasonably good agreement with the measured value of *μ*_e_ = 5900 cm^2^/(Vs), as has been reported in our photo-Hall measurement [37]. This indicates that the high electron mobility characteristics are maintained in our undoped GaAs transport layer, giving a significant contrast with the lowered mobility, as low as 200 cm^2^/(Vs) in the literature [47]. High electron mobility has been observed in a GaAs-based modulation-doped heterostructure PCA [58]. The density of states of the midgap states *N*_0_ = 2 × 10^16^ cm^−3^ is adopted from a previous work [37], and the electron density of states in the QDs is assumed to be given by the QD ground states *N*_Q_ = 3.4 × 10^15^ cm^−3^ referring to the measured QD density 1.7 × 10^15^ cm^−3^ [37]. The capture time *τ*_cT_ for electrons to the midgap states arising from defects such as EL2 is assumed to be 1 ps for InAs QDs in the GaAs transport layer [59]. The electron capture time to the empty QDs *τ*_cQ_ is assumed to be 0.5 ps, but a range from 0.2 ps to 2 ps [60,61,62] is considered for inspecting its effects in the following calculation. To evaluate the electron generation rate by Equation (4), the electron cross-section of the midgap states *σ*_TB_ = 3 × 10^−15^ cm^2^ as determined in our previous work [37] and initial density of unoccupied midgap states *n*_T0_ = 0.5 *N*_T_ are used, and the reflectivity of the PCA surface *R* = 0.318 is applied. The bias field *E*_b_ is set at 5 V/6 μm = 8.33 kV/cm. The effect of recombination time is assumed to be neglected in this analysis as already mentioned. The values of key parameters used in the calculation are summarized in Table 1.

## 4. Results and Discussion

**Figure 1:**
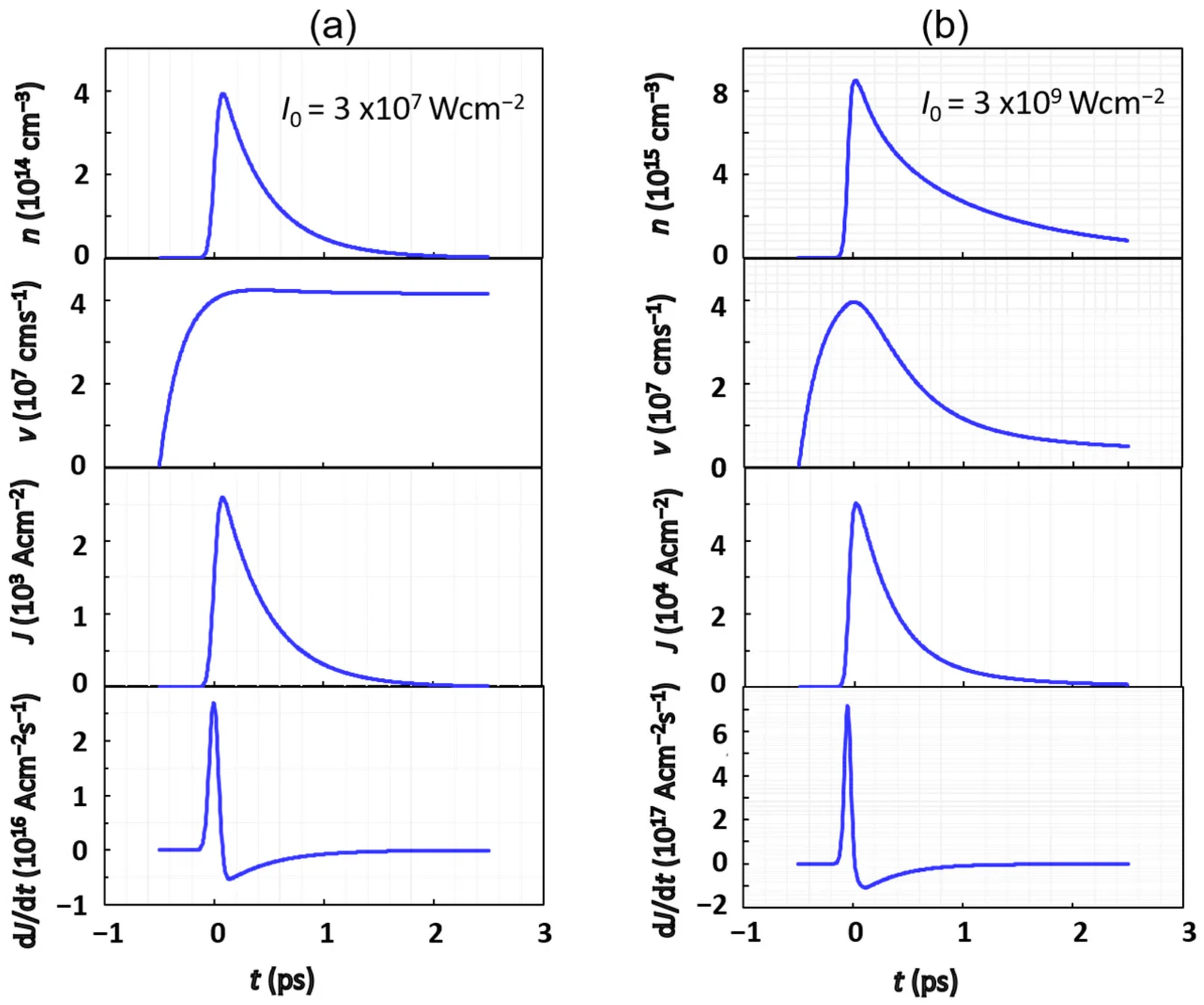
Calculated time dependences of the electron velocity (*v*), electron density (*n*), photocurrent density (*J*) and time derivative of photocurrent (d*J*/d*t*) at the excitation power density (**a**) 3 × 10^7^ Wcm^−2^ and (**b**) 3 × 10^9^ Wcm^−2^.

We first look at changes in the temporal behaviors of several key parameters for different excitation peak power density *I*_0_. Figure 1 illustrates the calculated results of the electron density, electron velocity, photocurrent density and time derivative of photocurrent density for two different excitation power densities: (a) 3 × 10^7^ Wcm^−2^ and (b) 3 × 10^9^ Wcm^−2^. The peak position of Gaussian excitation pulse is fixed at *t* = 0 in this series of calculations. At the lower excitation power density, as shown in Figure 1a, the photogenerated electron density shows a rapid rise followed by a decay caused by the capture by the midgap states and QDs, whereas the electron velocity after the pulse peak stays at a nearly constant level suggesting a nearly constant internal field applied to electrons. The electron mobility is assumed to be constant, as high as 5200 cm^2^/(Vs) and consistent with the experimental value [37], in this simulation. The photocurrent exhibits a decay curve resembling that of the electron density, reflecting the constant velocity. The current derivative shows a sharp positive peak at the leading edge of the excitation pulse followed by a slower negative peak after the excitation pulse. These peaks are thought to correspond, respectively, to the higher frequency and lower frequency components of the radiated THz field. In contrast, at 100 times higher excitation power density, as depicted in Figure 1b, the photogenerated electron density shows a hundred-times higher level, but its decay shows a decrease much slower than that in (a). The electron velocity behavior after the pulse excitation exhibits a considerably rapid decrease, implying a significant effect of space charge-induced retarding field in the transport layer. The photocurrent indicates a very rapid decrease, even faster than that shown in (a).

**Figure 2:**
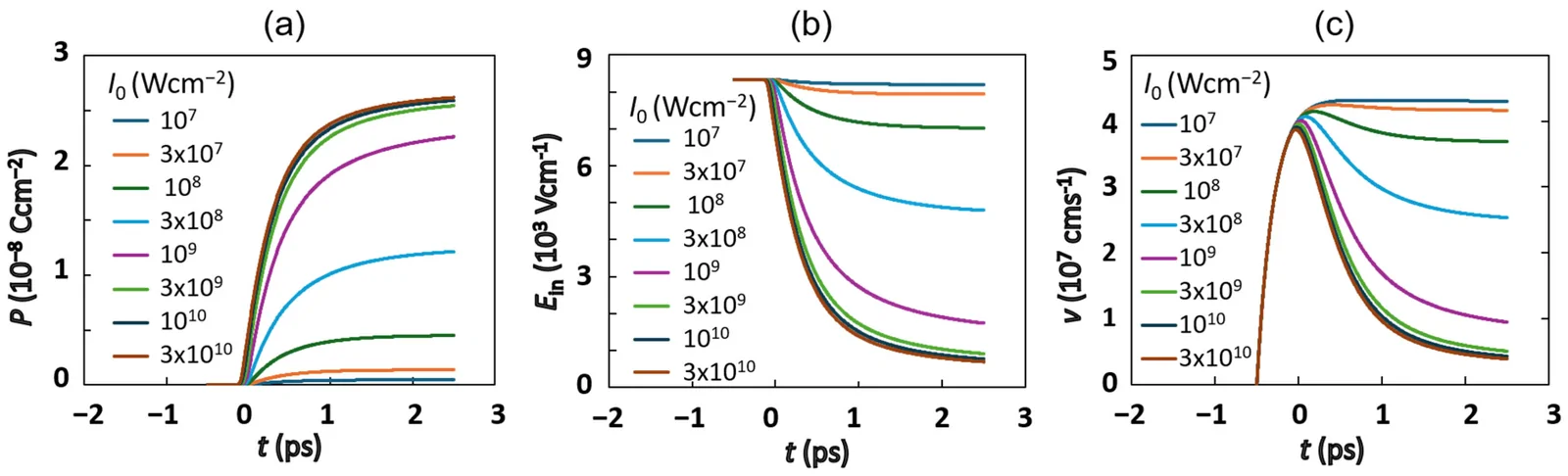
Time dependences of (**a**) the space charge polarization, (**b**) internal field and (**c**) electron velocity calculated for a variety of excitation power densities in the range from 1 × 10^7^ Wcm^−2^ to 3 × 10^10^ Wcm^−2^.

To understand the THz generation mechanism, we look at the temporal changes of the calculated parameters at different excitation power densities. Figure 2 displays time dependences of (a) the space charge polarization, (b) internal field and (c) electron velocity calculated for a variety of excitation power densities. The space charge polarization is immediately created as the photoelectrons are generated by the excitation pulse, the internal field is reduced by the retarding field due to space charge effect, and consequently the electron velocity is reduced. In the low-excitation power density range, the electron velocity increases to up to the level near the electron saturation velocity, reflecting the very high electron mobility maintained in the undoped GaAs-based transport layer. The electron velocity is kept at this high level as far as the carriers exist. The high mobility (>5200 cm^2^/(Vs)) maintained in the device is characteristic to the present PCA, quite different from the case in LT-GaAs PCAs [47]. Under high excitation levels, the internal field and therefore the electron velocity are heavily suppressed. The build-up of the polarization is basically limited by the momentum relaxation time, and the very fast decay behaviors of the internal field and electron velocity are reasonably understood. An extremely fast transient of the photocurrent as seen in Figure 1b is considered to be the consequence of the Coulomb screening effect.

**Figure 3:**
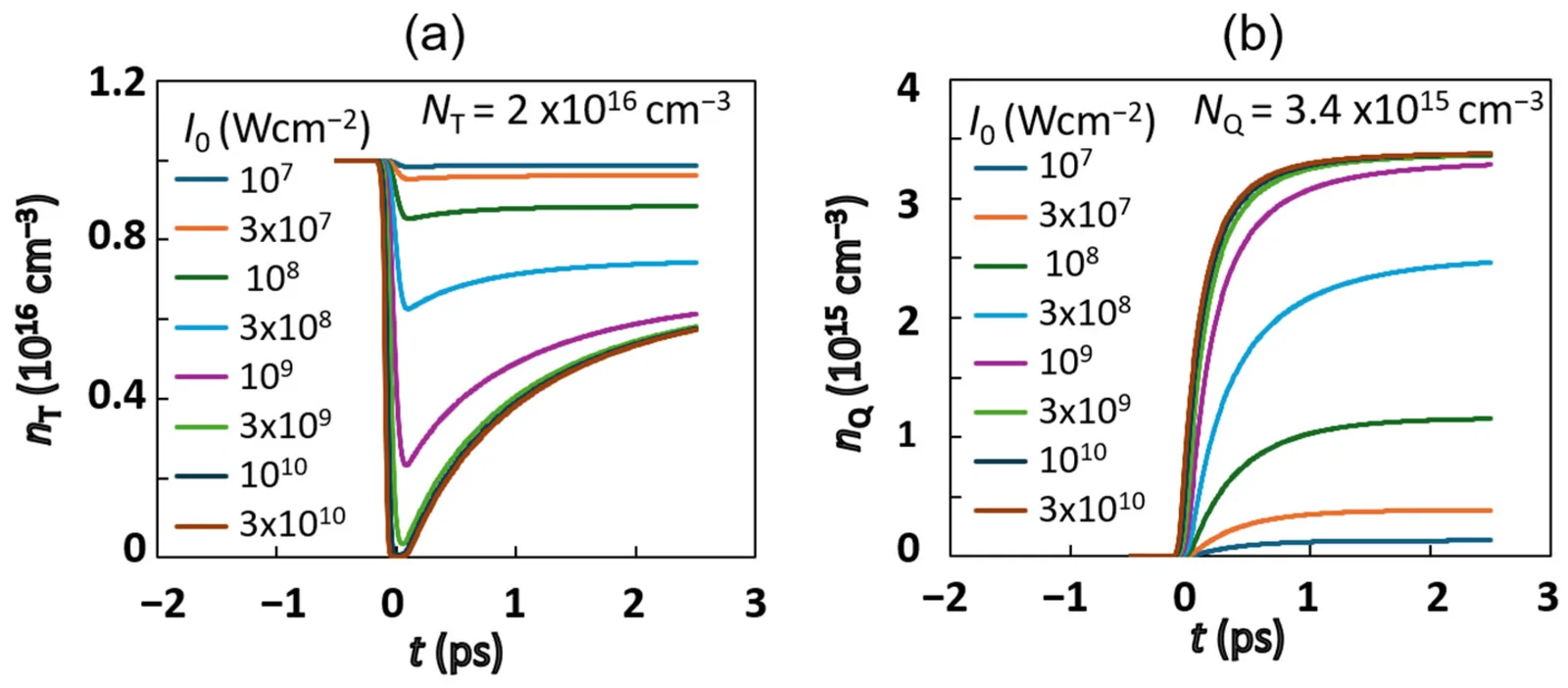
Time dependences of (**a**) the electron density occupying the midgap states *n*_T_ and (**b**) electron density captured in the QDs *n*_Q_ calculated for different excitation power densities in the range from 1 × 10^7^ Wcm^−2^ to 3 × 10^10^ Wcm^−2^. The initial conditions used are *n*_T_ = *N*_T_/2 and *n*_Q_ = 0.

Figure 3 shows the time dependences of (a) the electron density occupying the midgap states *n*_T_ and (b) electron density captured in the QDs *n*_Q_ calculated for different excitation power densities. As the initial conditions of this calculation, the midgap states were assumed to be half occupied by electrons, and the QDs were assumed to be initially empty. Electrons in the midgap states are excited by the laser pulse to the conduction band. The initial sharp drop of *n*_T_, as shown in Figure 3a, indicates this process. Then some of the conduction electrons are captured back into the midgap states by basically following the capture time constant. In the QDs, the capture of conduction electrons is restricted by the QD’s electron capture time as long as the conduction electron density is smaller than the QD density of states, but the electron capture to the QDs is much accelerated when the conduction electron density (*n*) exceeds the QD state density (*N*_Q_), as observed in Figure 3b.

**Figure 4:**
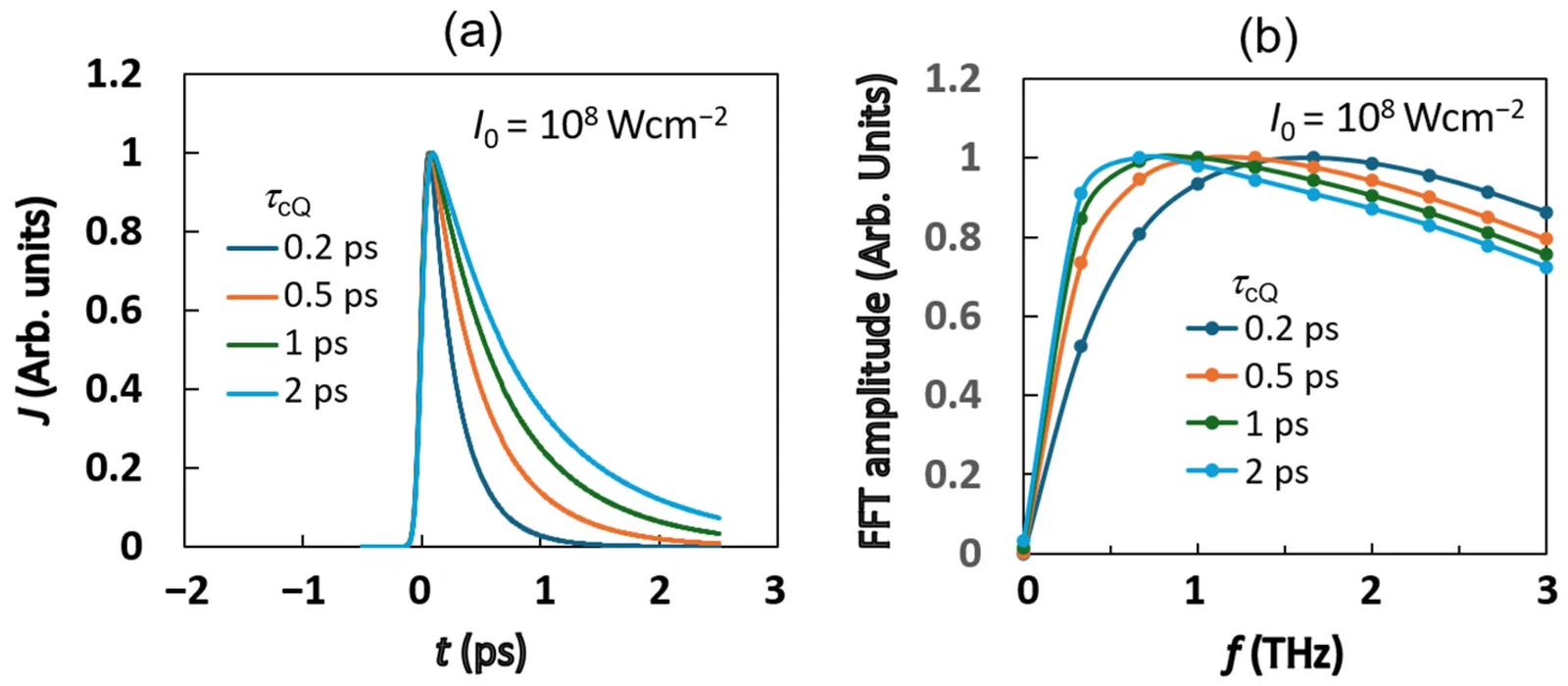
(**a**) The normalized current density as a function of time for the electron capture times of QDs *τ*_cQ_ in a range of 0.2–2 ps at the excitation power density *I*_0_ = 10^8^ Wcm^−2^. (**b**) The frequency dependences of the FFT amplitude of radiated THz field for the same *τ*_cQ_ values as those used in (**a**).

In the low excitation density range, the photocurrent decay curve is most directly influenced by the time for electron capture into the QDs. Figure 4a shows the normalized current density as a function of time for different electron capture times for QDs *τ*_cQ_ in a range of 0.2–2 ps at the excitation power density *I*_0_ = 10^8^ Wcm^−2^. The radiated THz field was calculated from d*J*/d*t* by using the fast-Fourier transform (FFT) program, and its normalized value is displayed in Figure 4b. The peak THz field frequency decreases as *τ*_cQ_ increases, and the peak frequency of ~1 THz is obtained when *τ*_cQ_ = 0.5 ps as adopted in the rest of the present calculation. In the higher excitation power range, all the QDs become full at the early stage of photoexcitation, and the photocurrent decay transient is determined predominantly by the effects of the conduction electron density and the space charge-induced electron velocity, eventually resulting in the photocurrent decay time being even faster than the QD capturing time, as observed in Figure 1b.

**Figure 5:**
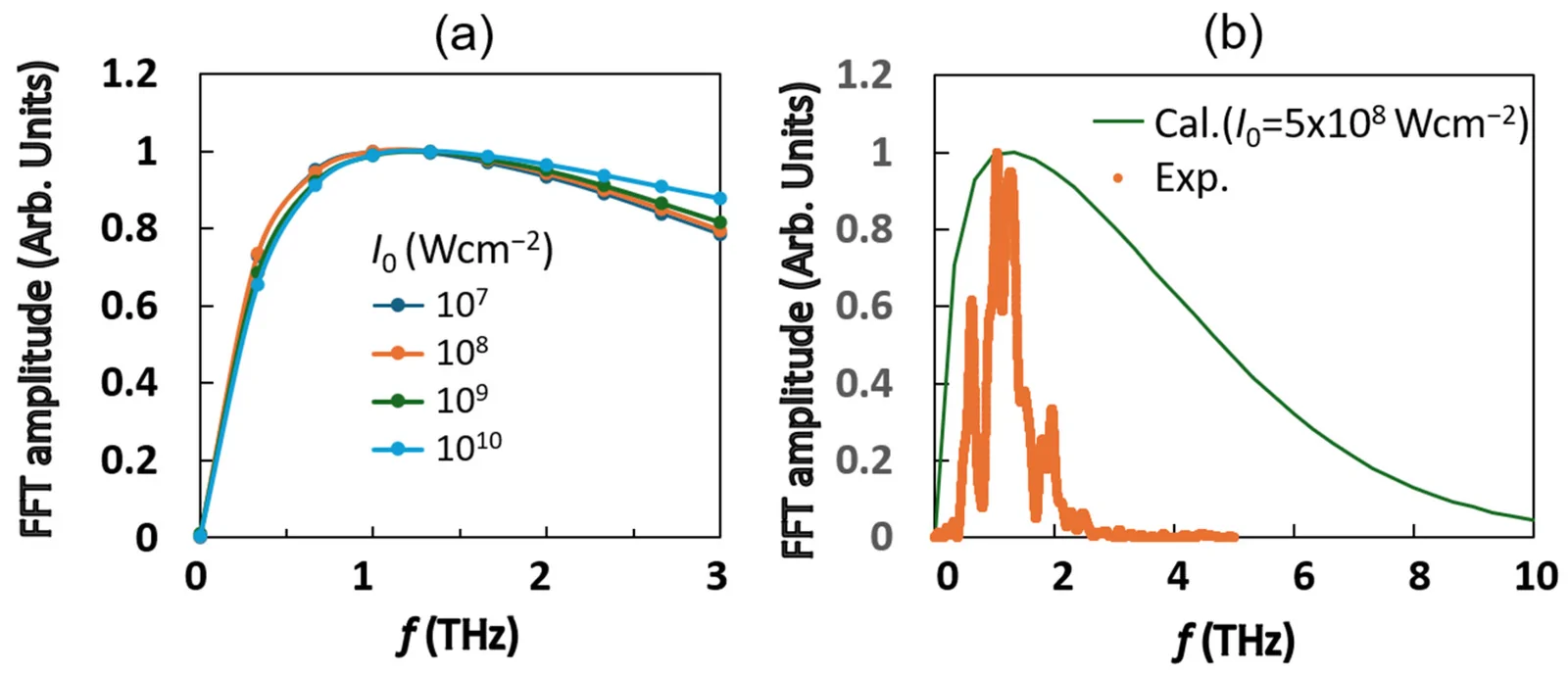
(**a**) THz field FFT amplitude spectra calculated for different excitation power densities: *I*_0_ = 10^7^–10^10^ Wcm^−2^. (**b**) Radiated THz field FFT amplitude spectrum calculated for the excitation power density of 5 × 10^8^ Wcm^−2^ and the experimental spectrum at the same excitation power density taken from our previous work [36].

In Figure 5a, the THz field FFT amplitude spectra calculated for different excitation power densities in the range of *I*_0_ = 10^7^–10^10^ Wcm^−2^ are shown. The THz peak frequency is observed to slightly increase only when the excitation power is maximized. Figure 5b displays an expanded view of the spectrum calculated for the excitation power density of 5 × 10^8^ Wcm^−2^, and an experimental spectrum taken at the same excitation power density in our previous work [36] is also included. The experimental curve involves significant dips due to the water vapor absorption peaks (e.g., 0.38, 0.57, 0.75, 1.2-1.4 THz, etc. [63]) and a more serious drop in the high frequency range. To understand the physical origin of the high frequency drop, the influence of the frequency characteristics of antenna was examined. The half-wavelength dipole antenna used in our PCA device has a dipole length of 20 μm, an electrode width of 10 μm, and a stripline width of 10 μm. The effective dipole length (*l*_e_) determined from these dimensions is 41 μm, and the resonance frequency of the antenna (*f*_ant_) is calculated by *f*_ant_ = c/{2*l*_e_[(1 + ε)/2]^1/2^} [64] to be 1.4 THz. It is also known that the dipole antenna has a relatively narrow passband width (e.g., ~20% of *f*_ant_ or narrower by special design [65]). A recent study on GaAs-based PCAs with similar dipole lengths reported a peak THz emission frequency as low as 0.4 THz, implying considerable narrowing of the THz spectra by the antenna filtering effect [66]. By presuming the resonance frequency in our case to be approximately 1 THz, we can attribute the discrepancy in the spectrum in Figure 5b to the antenna filtering effect. In addition, the detector used in our experiment is LT-GaA-based PCA with a dipole antenna with similar geometry, and this is considered to enhance the filtering effect, leading to an even sharper THz emission spectrum. This interpretation would reasonably explain the observed difference between the theoretical and experimental THz spectra in Figure 5b. Similar narrow-width THz spectra have been observed in LT-GaAs PCAs [59,66].

**Figure 6:**
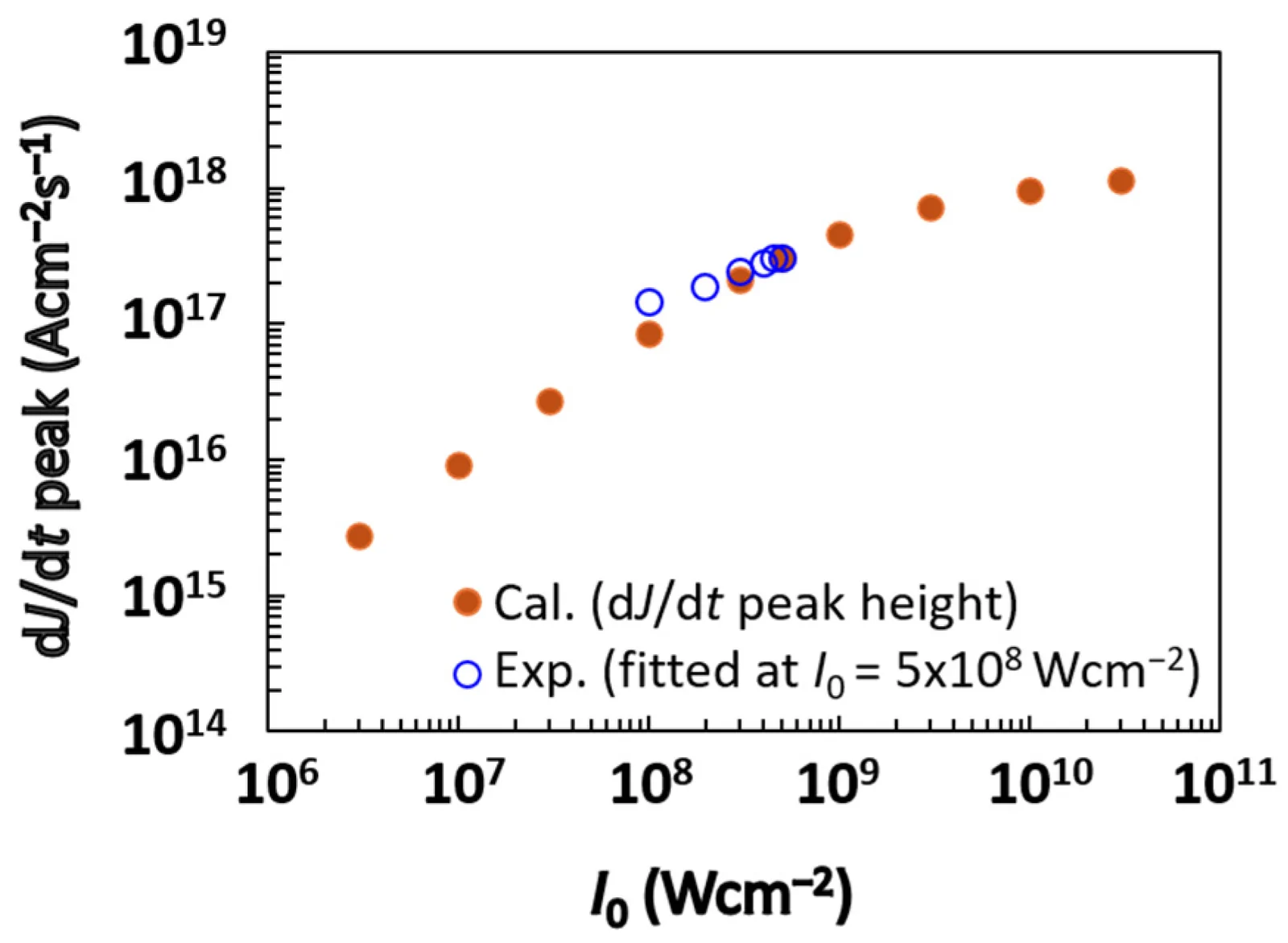
Relationship of the radiated THz field amplitude as a function of the excitation power density. The THz field amplitude is represented by the peak height of the positive d*J*/d*t* peak. Experimental data were taken from a previous paper and fitted with the calculation at the excitation power density of 5 × 10^8^ Wcm^−2^ [35].

Figure 6 shows the relationship of the radiated THz field amplitude as a function of the excitation power density. Considering that the radiated THz field amplitude is related with d*J*/d*t*, we use the peak height of d*J*/d*t* for representing the THz field amplitude. As seen in Figure 6, the THz field amplitude tends to saturate as the excitation power density increases. Such a saturation behavior is not seen when the photocurrent is proportional to the bias field (*J* = *enμ*_e_*E*_b_). This saturation occurs because the internal field is reduced by the Coulomb space-charge screening effect. To distinguish the contributions of the Coulomb screening and THz field screening, a test to determine the photocurrent by *J* = *enμ*_e_*E*_in_ instead of Equation (9) was conducted at *I*_0_ = 1–3 × 10^9^ Wcm^−2^. It was found that the THz field screening effect contributes by only several percent to the total reduction in the d*J*/d*t* peak height from the unscreened value, indicating that THz saturation behavior is predominated by the Coulomb space charge screening effect in the present device. The radiated THz field screening effect has been reported to become significant particularly when a large aperture PCA is considered, where the near-field component of the radiated THz emission has a parallel but opposite field against the bias field [53]. The observed minor role of the THz field screening is consistent with our PCA having a narrow gap structure.

A set of experimental data shown in Figure 6 includes the photocurrents measured in an Er-doped InAs QD-containing InGaAs PCA, which is the only excitation power dependence data reported [35]. Considering that the FWHM of the negative d*J*/d*t* peak (corresponding to the sub-THz spectrum) does not vary substantially for the excitation power change, the measured photocurrent corresponding to the integrated d*J*/d*t* value can be used for comparison with the present calculation. The calculated and measured photocurrents have been fitted at the excitation power density 5 × 10^8^ Wcm^−2^, corresponding to the excitation power density used in the experiment [35]. The slopes of the calculated and experimental data are similar, but the agreement is not sufficient to fully support the present calculation model. This discrepancy may arise from the substantial difference in the PCA structure, including the host material (InGaAs) and, in particular, its bandgap energy. At a wavelength of 1550 nm, the absorption in InGaAs is much higher (by approximately one to two orders of magnitude) than that associated with midgap state excitation in GaAs. This difference effectively shifts the experimental data points in Figure 6 toward the higher excitation power density region, which may account for the limited agreement between the calculated and experimental results. Nevertheless, THz power saturation predominantly caused by Coulomb screening has been widely observed in other PCAs [27,43,59,64], suggesting that a similar mechanism is likely to operate in the present QD-containing GaAs-based PCAs.

**Figure 7:**
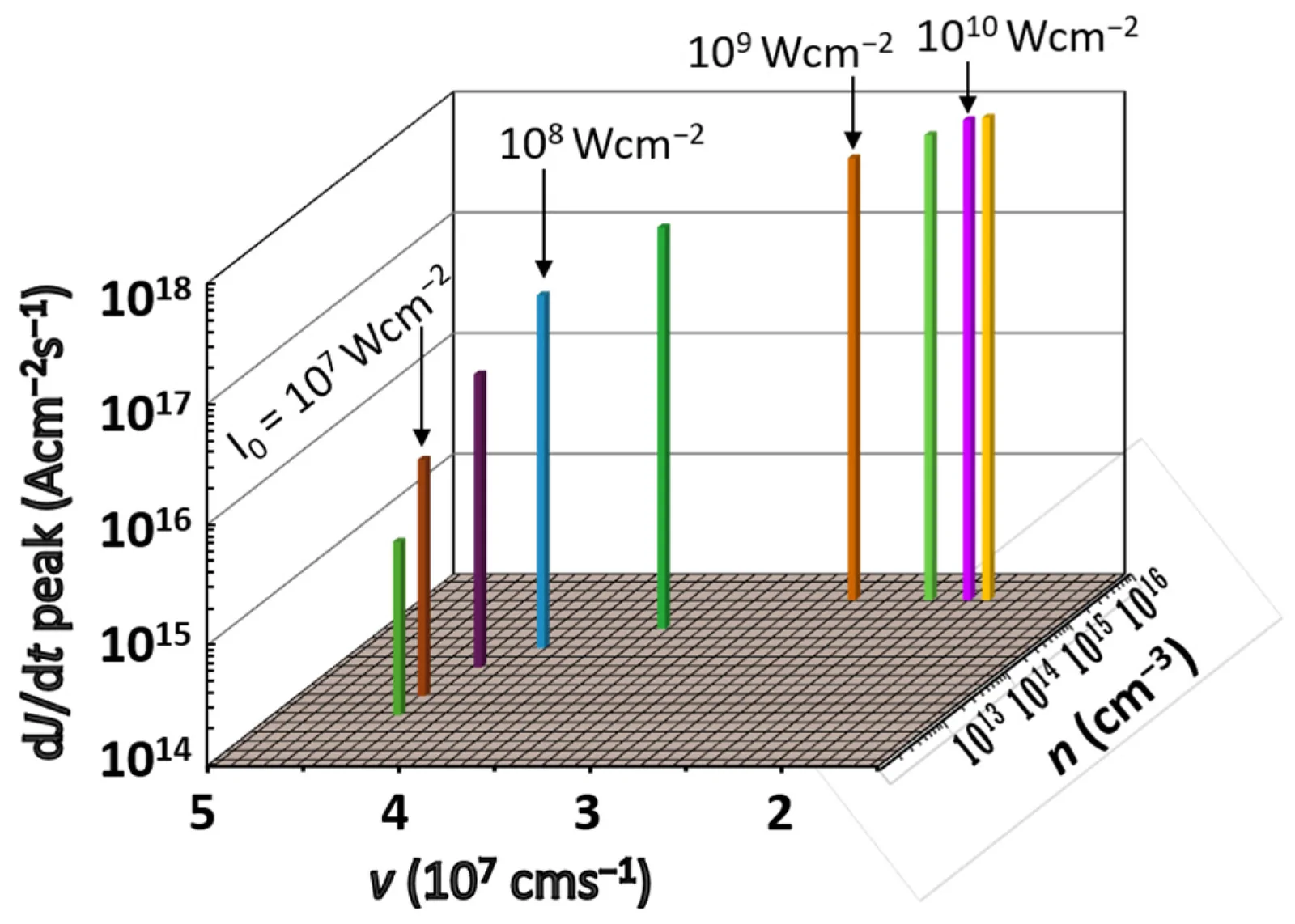
Three-dimensional representation of the positive current derivative peak height (d*J*/d*t*) which corresponds to the peak radiated THz field amplitude, the conduction electron density (*n*), and the electron velocity (*v*), all determined at the time *t* = 0.5 ps for different photoexcitation power density (*I*_0_) levels in the range from 3 ×10^6^ to 3 × 10^10^ Wcm^−2^. Colors of data bars only show the difference of *I*_0_.

For grasping an overall trend of the THz generation mechanism in those PCAs, three key parameters, the positive current derivative peak height (d*J*/d*t*), conduction electron density (*n*) and electron velocity (*v*), all of which are determined at the sub-ps time (*t* = 0.5 ps), are visualized in a three-dimensional map in Figure 7. When the excitation power is in the lower range, the THz generation is caused by the electron density transient, and the high electron velocity is maintained. In this situation, QDs are not fully filled with electrons, and the THz spectrum profile is determined by the capture time of electrons to fall into the QDs. As the excitation power increases to the middle range, QDs become saturated with electrons; meanwhile, the Coulomb screening causes a reduction in electron velocity, resulting in an even faster current transient and THz field radiation with enhanced high frequency components. As the excitation level further increases, the Coulomb screening effect predominantly limits the radiation of THz field and causes severe reduction in the electron velocity and radiated THz field amplitude. The effects of electron velocity transient/saturation under high electric fields [48,67] have not been incorporated into our calculation model. According to a Monte Carlo simulation study of ballistic electron transport in GaAs [68], the electron velocity initially overshoots to approximately 4 × 10^7^ cm/s and then rapidly decreases over a time scale of 1–2 ps, eventually approaching a steady-state value of approximately 2 × 10^7^ cm/s under the electric field of ~8 kV/cm. Although the present model does not include the velocity overshoot effect, the results shown in Figure 7 appear to be consistent with the realistic transient behavior of electron velocity under a high electric field. A more precise evaluation of the electron velocity transient under high-field conditions requires further investigation.

**Figure 8:**
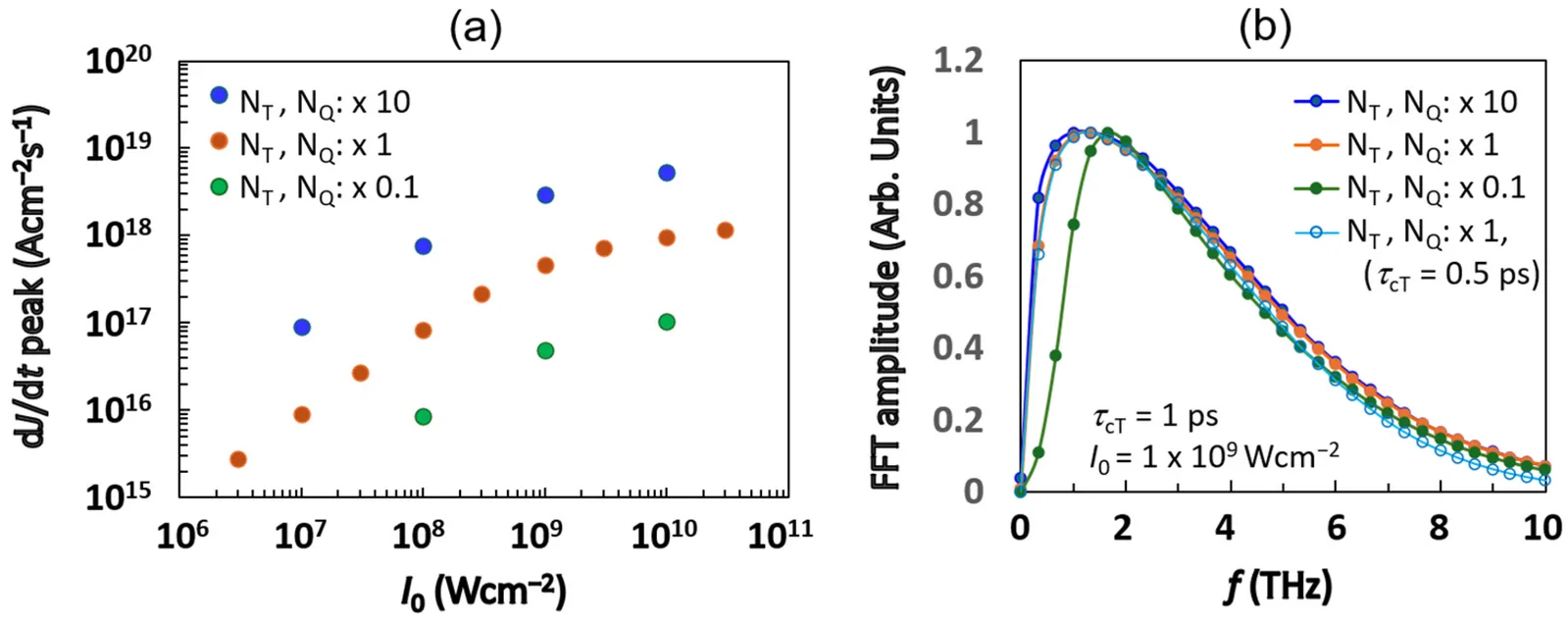
(**a**) Relationships of the positive d*J*/d*t* peak height as a function of the excitation power density for different sets (×10, ×1, ×0.1) of *N*_T_ and *N*_Q_ values with the *N*_T_/*N*_Q_ ratio maintained constant. (**b**) Radiated THz field FFT amplitude spectra calculated for different sets (×10, ×1, ×0.1) of *N*_T_ and *N*_Q_ values with the *N*_T_/*N*_Q_ ratio maintained constant under the excitation power density *I*_0_ = 1 × 10^9^ Wcm^−2^ and the electron capture time of midgap states *τ*_cT_ = 1 ps. Another curve contained shows the spectrum when *τ*_cT_ is changed to 0.5 ps under the standard (×1) values of *N*_T_ and *N*_Q_.

We have so far presented and discussed the results using a selected set of physical parameter values. It is therefore worthwhile examining how variations in these parameters and their uncertainties affect the results, both for understanding the underlying physics and for practical device design. Figure 8a shows the dependence of the THz radiation field on the excitation power density when the midgap-state density (*N*_T_) and QD density of states (*N*_Q_) are both increased by a factor of 10 or decreased by a factor of 10 relative to the primarily adopted values of *N*_T_ = 2 × 10^16^ cm^−3^ and *N*_Q_ = 3.4 × 10^15^ cm^−3^. The ratio of *N*_T_ to *N*_Q_ was kept constant for simplicity. As shown in the figure, the overall dependence of the THz field amplitude on the excitation power density is relatively similar among the three cases, except for the enhanced saturation observed above *I*_0_ = 1 × 10^9^ Wcm^−2^ when both *N*_T_ and *N*_Q_ are increased by a factor of 10. Figure 8b shows the corresponding FFT amplitude spectra for the same variations in *N*_T_ and *N*_Q_. The spectra exhibit only minor changes, except for the case of a high midgap-state density (10 times the standard value) combined with a high excitation power density (>1 × 10^9^ Wcm^−2^). The enhanced saturation of the THz amplitude and the distortion of the THz spectrum under this extreme condition imply an enhanced contribution from THz field screening. A detailed investigation of this effect is beyond the scope of the present study and is subject to future work. Recent studies have reported detailed formalisms and simulation results for screening effects in PCAs, as well as approaches to suppress space charge screening and improve THz emission efficiency through nanostructure engineering [69,70].

In Figure 8b, we also show a spectrum calculated with a reduced electron capture time for the midgap states, *τ*_cT_ = 0.5 ps, instead of the primarily adopted value of 1 ps, using the primarily adopted values of *N*_T_ and *N*_Q_. The overall spectral shape remains almost unchanged, except for a slight reduction in the high-frequency component compared with the *τ*_cT_ = 1 ps case. This result indicates that the spectrum shape is relatively insensitive to the *τ*_cT_ under these excitation conditions. At first glance, this behavior appears different from the influence of *τ*_cQ_ on the spectrum shown in Figure 4a. However, at an excitation power density of 10^8^ Wcm^−2^ (Figure 4a), the QDs are not yet fully occupied, and the photocurrent transient is therefore strongly affected by the QD capture time. In contrast, at an excitation density of 10^9^ Wcm^−2^ (Figure 8b), the QDs are already substantially occupied, and the decay of the conduction electron density becomes slower (see the upper panel of Figure 1b). Under these conditions, the effect of shortening the midgap state capture time on the photocurrent transient and THz spectrum is likely to be suppressed.

As described above, the simplified model developed in this work for the dynamic behavior of photocurrent in QD-containing GaAs-based PCAs has revealed the roles of QDs and their interplay with photocarrier generation from midgap states, carrier capture by QDs, and space charge- and radiated THz field-induced screening in the THz wave-generation process. In the low-excitation power region, electron capture by the QDs strongly influences the photocurrent transient and the THz field spectrum. In the high-excitation power region, Coulomb space charge screening causes saturation of the THz field amplitude, whereas the THz field spectrum is relatively insensitive to variations in the key parameters.

These results also provide several implications for practical device design. A short electron capture time in the QDs is most effective in modifying the photocurrent transient and THz field amplitude in the relatively low-excitation power region, as long as the QD states are not fully occupied. To maintain this rapid carrier capture effect over a wider range of excitation power, the QD density could be increased toward the density of the midgap states. In addition, increasing the midgap state density is expected to increase the saturation level of the THz field amplitude. However, an excessive increase in the density of midgap states and/or QDs may adversely affect the electronic properties of the material, for example by reducing the electron mobility. These trade-offs should therefore be considered carefully in material and device optimization.

## 5. Conclusions

The THz wave-generation mechanism in InAs QD-containing GaAs-based PCAs was analyzed by numerical simulation using a simplified rate equation model. The model assumes two-step photoexcitation through midgap states and rapid carrier capture into the QDs. Temporal variations in the key physical parameters were simulated under 1550 nm photoexcitation. At low excitation power, the photocurrent and radiated THz field amplitude are primarily governed by the photogenerated electron density, while the electron velocity remains at a level determined by the electron mobility and applied bias field. The photocurrent transient and radiated THz spectrum are strongly influenced by the electron capture time into the QDs. As the excitation power increases, the internal electric field is reduced by the retarding field associated with Coulomb space charge screening, resulting in a substantial reduction in the electron velocity and photocurrent. At higher excitation power, the THz field amplitude becomes saturated primarily because of Coulomb space charge screening, while the contribution of radiated THz field screening remains limited for the present device structure. The influence of variations in several key parameters on the THz generation characteristics was also examined, providing basic implications for device optimization. The results indicate that the QD capture time and density are particularly important in the low-excitation power region, whereas the midgap state density affects the saturation behavior at high excitation power. Excessive increases in the densities of midgap states and QDs, however, may adversely affect the electronic properties of the material, indicating a trade-off between carrier dynamics and material quality. The device characteristics revealed by this analysis are specific to QD-containing GaAs-based PCAs and may contribute to a better understanding of the underlying device physics and to the optimization of practical THz source structures.

## Figures and Tables

**Table 1:** Table of the values of key parameters used in this calculation and source references.

Parameter	Value	Reference
Electron scattering time (*τ*_s_)	200 fs	[37]
Electron mobility (*μ*_e_)	5200 cm^2^/(Vs)	[37]
Midgap state density (*N*_0_)	2 × 10^16^ cm^−3^	[37]
QD density of states (*N*_Q_)	3.4 × 10^15^ cm^−3^	[37]
QD electron capture time (*τ*_cQ_)	0.5 ps (0.2–0.5 ps)	[37] ([60,61,62])
Midgap state electron capture time (*τ*_cT_)	1.0 ps	[37,59]
Midgap state electron cross-section (*s*_TB_)	3 × 10^15^ cm^2^	[37]
Bias field (*E*_b_)	8.33 kV/cm	[36]
Excitation wavelength (*λ*)	1550 nm	[36]
Excitation pulse width (FWHM)	100 fs	[36]
Excitation pulse repetition rate (*f*_rep_)	80 MHz	[36]

## Data Availability

The raw data supporting the conclusions of this article will be made available by the authors on reasonable request.
